# Bilateral recurrent laryngeal nerve paralysis diagnosed using dynamic digital radiography during the COVID‐19 pandemic

**DOI:** 10.1002/ccr3.6124

**Published:** 2022-07-25

**Authors:** Yukimi Shibuya, Koichi Hirano, Haruhiko Machida, Makoto Miyamoto, Kozue Watabe, Tomoya Mitsuma, Yoko Nakazato, Keisei Tachibana, Ryota Tanaka, Haruhiko Kondo

**Affiliations:** ^1^ Department of Thoracic and Thyroid Surgery, Faculty of Medicine Kyorin University Tokyo Japan; ^2^ Department of Radiology, Faculty of Medicine Kyorin University Tokyo Japan; ^3^ Department of Radiology Tokyo Women's Medical University Adachi Medical Center Tokyo Japan; ^4^ Department of Otolaryngology‐Head and Neck Surgery, Faculty of Medicine Kyorin University Tokyo Japan

**Keywords:** bilateral recurrent laryngeal nerve paralysis, COVID‐19, dynamic digital radiography, esophageal cancer, thyroid surgery

## Abstract

Dynamic digital radiography (DDR) is a motion‐detecting technique with high temporal resolution. Flexible laryngoscopy is a common modality for the observation of the larynx; however, it generates aerosol. DDR is an easy and less risky screening test for the diagnosis of recurrent laryngeal nerve paralysis during the COVID‐19 pandemic.

## Graphical abstract

Dynamic digital radiography (DDR) is a motion‐detecting technique with high temporal resolution. It is a useful tool to assess vocal cord's motion, and can be an alternative modality to flexible laryngoscopy, which could cause an aerosol exposure to examiners.

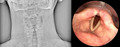

## 1 INTRODUCTION

Flexible laryngoscopy (FL) is a common diagnostic modality for recurrent laryngeal nerve paralysis (RLNP). During FL, healthcare workers, particularly the operator and assistants, are at a substantial risk of coronavirus disease (COVID‐19) transmission because it requires frequent, prolonged exposure to and manipulation of the aerodigestive tract mucosa, where viral loads are the highest.^1^ Additionally, it is potentially an aerosol‐generating procedure.^2^

Dynamic digital radiography (DDR) is a state‐of‐the‐art radiographic technique with high temporal resolution (15 frames/second) using an optimized flat‐panel detector (FPD).^3^ This FPD offers fluoroscopy‐like images of sufficient quality at a low total radiation dose. Although this modality has never been applied in laryngeal radiography, this examination is easy, cost‐effective, non‐invasive, and can null the risk of COVID‐19 transmission. Thus, we applied DDR to our patients with bilateral RLNP (BRLNP). BRLNP is a serious and life‐threatening clinical condition resulting from various causes (e.g., invasion of malignant tumors and injury during head and neck surgery). Patients with BRLNP often require immediate and appropriate treatment, such as early tracheal intubation and tracheostomy, to avoid asphyxia and severe aspiration. Herein, we present the first two cases of patients with BRLNP who were successfully diagnosed using DDR, without the risk of COVID‐19 transmission, as a screening test. Then, they were successfully managed using early tracheostomy.

## 2 CASE REPORTS

### 2.1 Case 1

**FIGURE 1:**
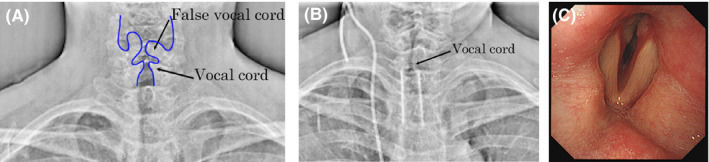
Dynamic digital radiography (DDR) during deep inspiration on day one (A) and day nine (B) and bronchoscopy during phonation on day nine (C) in Case 1. DDR on day one (A) shows paramedian fixation of the left vocal cord, and the bilateral vocal cords on day nine (B), as confirmed using bronchoscopy on the same day (C).

A 54‐year‐old woman was referred to our hospital with chief complaints of hoarseness, stridor, and severe dyspnea. Owing to her poor condition, FL would have been difficult to perform; thus, she underwent DDR examination of the neck and chest during deep breathing at a low total radiation dose of 1.16 mGy. Paramedian fixation of her left vocal cord was revealed (Figure 1A and Video S1). She further underwent contrast‐enhanced neck and chest computed tomography (CT) that disclosed upper‐to‐middle thoracic esophageal cancer, mediastinal and bilateral cervical lymph node metastases, and right lung metastasis. Some of the cervical lymph node metastases had invaded both the thyroid lobes and the trachea; in particular, some of the left cervical lymph node metastases were considered to have caused the left RLNP. Following tracheal intubation, a tracheal stent was placed using a Dumon™ stent (Novatech, Aubagne, France) to relieve dyspnea due to severe tracheal stenosis on day three of hospitalization. After placement of the tracheal stent, she experienced discomfort in the throat and, thus, underwent repeated DDR examination on day nine of hospitalization, which revealed bilateral paramedian fixation of her vocal cords (Figure 1B and Video S2). To avoid asphyxia, tracheal intubation was performed under bronchoscopy on the same day, which further confirmed this finding (Figure 1c). The patient underwent tracheostomy on Day 17 of hospitalization. Thereafter, the tumors continued to progress and were refractory to chemoradiotherapy. However, the patient did not experience asphyxia or severe aspiration as a result of BRLNP until she died owing to severe intratracheal tumor bleeding on Day 149 of hospitalization.

### 2.2 Case 2

**FIGURE 2:**
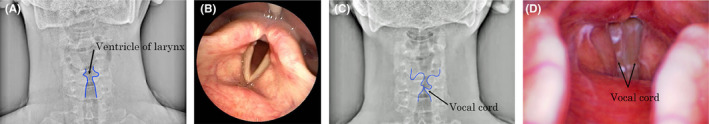
Dynamic digital radiography (DDR) (A, C) and flexible laryngoscopy during phonation (B, D) on day three (A, B) and Day 72 (C, D) in Case 2. DDR on day three (A) shows intermediate fixation of the bilateral vocal cords, as confirmed using flexible laryngoscopy on the same day (B). Both DDR (C) and flexible laryngoscopy on Day 72 (D) show complete closure of the bilateral vocal cords.

A 20‐year‐old man with triiodothyronine‐predominant Graves' disease refractory to medical treatment was referred to our hospital for thyroid surgery. The patient underwent total thyroidectomy through a collar incision on day two of hospitalization. During the dissection of the left thyroid lobe from the surrounding tissues, the left RLN was confirmed to be preserved both visually and functionally using intraoperative neuromonitoring (IONM). However, dissection of the right thyroid lobe was difficult because of its adhesion to the surrounding tissues; thus, the right RLN was accidentally injured. The proximal end of the right RLN was anastomosed with the right cervical ansa. Finally, the left RLN was reconfirmed to be functionally preserved using IONM before closure of the incision. The gross thyroid specimen measured 11 × 10.5 × 5 cm^3^ and weighed 197 g. The patient presented with severe hoarseness on day three (i.e., postoperative day two [POD 2]), and on day four (POD 3), he underwent DDR examination of the neck during phonation at a low total radiation dose of 1.15 mGy; it revealed bilateral intermediate fixation of his vocal cords (Figure 2A and Video S3). On the same day, BRLNP was confirmed using FL (Figure 2B), and the patient underwent tracheostomy to avoid asphyxia. The patient was uneventfully discharged on Day 44 of hospitalization (POD 43). His hoarseness was markedly relieved by Day 65 (POD 64). He underwent both the DDR examination (Figure 2C and Video S4) and FL (Figure 2D) again, which showed remarkably improved movement of his left vocal cord; therefore, tracheostomy was discontinued on Day 72 (POD 71).

During the DDR examination, both patients wore a mask, and the radiographers wearing a surgical mask touched them for patient positioning only for less than 20 s and maintained at least 1 m distance otherwise.

## 3 DISCUSSION

Although FL is a common diagnostic modality for RLNP, it is potentially an aerosol‐generating procedure that has garnered widespread caution as a high‐risk procedure for COVID‐19 transmission.^2^ Thus, recommendations from national and international health organizations suggest minimizing its use.^4^, ^5^ FL is also associated with patient discomfort, intolerance to its insertion, and the need for ear–nose–throat (ENT) specialists with sufficient experience and skills. This examination can be limiting for patients with a severe gag reflex and/or unfavorable anatomical features of the larynx.^6^

Dynamic digital radiography is a novel functional imaging technique that uses the FPD radiography system and provides temporally resolved images of various anatomies. Chest DDR has been used to analyze diaphragmatic kinetics and lung ventilation/perfusion.^3^ DDR is advantageous over FL in being easier, more cost‐effective, and non‐invasive, without the necessity of ENT specialists or any risk of COVID‐19 transmission. Thus, we performed DDR as a screening test and successfully diagnosed BRLNP in both our cases. However, DDR has never been applied for this purpose. As in our cases, patients with BRLNP require appropriate treatment, such as early tracheal intubation and tracheostomy, to avoid asphyxia and severe aspiration. We believe that this is the first report regarding the clinical usefulness of DDR for the accurate diagnosis and appropriate management for patients with RLNP. In patients with unilateral RLNP who have symptoms such as hoarseness and dysphonia, as in Case 1, a change in their symptoms can be overlooked at the onset of BRLNP. DDR is particularly useful for diagnosing BRLNP in such patients owing to lower barriers to clinical implementation than those for FL.

Recurrent laryngeal nerve paralysis is one of the most common complications following surgeries in the head and neck region, such as thyroid/parathyroid surgery, for which the reported incidence of RLNP is 14%.^7^ Even though RLN injuries are not visually recognized intraoperatively, unexpected RLNPs can still occur, as in Case 2.^7^, ^8^, ^9^ Patients without RLNPs can experience discomfort in the throat and vocal alterations after these operations.^10^ Following up on patients who have undergone head and neck surgeries using DDR is more useful for diagnosing unexpected RLNP and excluding RLNP because of the easier clinical implementation than that for FL. Furthermore, DDR allows easy, non‐invasive, temporally successive monitoring of the recovery process of RLNP, as in Case 2. This case demonstrated the efficacy of DDR as a new diagnostic modality for RLNP.

In Case 1, posteroanterior DDR neck and chest images were acquired as part of screening conducted during the patient's deep breathing for approximately 10 seconds in the standing position. The first DDR examination incidentally disclosed the left RLNP. Based on this clinical experience, in Case 2, the DDR acquisition protocol was modified to improve the visual assessment of vocal cord movement. Specifically, using a reference line (i.e., acanthiomeatal line), anteroposterior DDR images of the neck with a smaller field of view were acquired during the patient's phonation and deep inspiration, with his head fixed by a fixture used in head radiography and chin raised to 10 degrees for approximately 10 s in the sitting position. The latter protocol can decrease susceptibility to capturing artifacts associated with the patient's motion, improve spatial resolution, increase the moving distance of the vocal cords during their opening and closing, and, thus, improve delineation of vocal cord movement. Although DDR differs from FL in requiring radiation exposure, the total radiation dose in each DDR examination was lower than the dose limit for two projections (posteroanterior and lateral views) during chest radiography as recommended by the International Atomic Energy Agency (i.e., 1.9 mGy).^11^ During the DDR examination, as in our cases, the patient does not need to remove the mask and/or face shield, and a single radiographer wearing adequate, but not heavy, personal protective equipment only needs to touch the patient for a very short period of time. Otherwise, they can maintain sufficient distance from the patient, significantly reducing the risk of COVID‐19 transmission when compared with that caused by FL. Thus, the DDR examination to diagnose RLNP is considered a very‐low risk procedure. For patients with suspected or confirmed COVID‐19, DDR may be clinically useful as a screening test to diagnose RLNP; in this scenario, FL should only be performed when DDR is inconclusive.

Other modalities to diagnose RLNP include laryngeal ultrasonography, fluoroscopy, CT, magnetic resonance imaging (MRI), and dual‐energy subtraction (DES) radiography. Although laryngeal ultrasonography is a non‐invasive test that does not need radiation exposure, its diagnostic accuracy is limited and highly dependent on the examiner's skill. The presence of medical devices, such as drainage catheters, and hematomas can limit the acoustic window and lead to misdiagnosis in patients following head and neck surgeries.^12^ The diagnostic performance of laryngeal fluoroscopy is inferior to that of DDR because of its higher radiation dose and poorer image quality. The spatial and temporal resolutions of CT and MRI are much lower than those of DDR. MRI examinations involve lower accessibility, longer examination time, and greater medical costs. Greater radiation exposure by CT and more prominent motion and susceptibility artifacts on MRI are also problematic. DES radiography uses two different energy X‐ray exposures and produces an image of only soft tissues by exploiting the difference between the energy‐dependent attenuation of bones and soft tissues to eliminate bone contrast. Laryngeal DES radiography allows easy visual evaluation of the vocal cord during phonation and inspiration by eliminating the cervical spine opacity.^13^ In patients with unilateral RLNP, the lack of movement of the paralyzed vocal cord when compared with the normal side can be visualized by comparing these static radiographs acquired during phonation and inspiration.^13^ For patients with BRLNP whose vocal cord movement can be more symmetrically impaired, laryngeal DDR, owing to its temporally resolved nature, has a higher sensitivity in diagnosis than DES radiography. We are currently developing a system to quantify vocal cord movement using laryngeal DDR. Further optimization of the DDR acquisition protocol can reasonably reduce radiation exposure and improve the diagnostic accuracy for RLNP.

## 4 CONCLUSION

This is the first report to demonstrate that DDR at a low radiation dose is clinically useful as an easy and non‐invasive screening test for accurate diagnosis and adequate management of BRLNP with almost no risk of COVID‐19 transmission.

## AUTHOR CONTRIBUTIONS

Yukimi Shibuya, Koichi Hirano, and Haruhiko Kondo conceived the presented idea. Yukimi Shibuya, Kozue Watabe, Tomoya Mitsuma, Yoko Nakazato, Keisei Tachibana, and Makoto Miyamoto collected the data. Yukimi Shibuya wrote the manuscript with support from Haruhiko Machida. All authors discussed the results and contributed to the final manuscript.

## FUNDING INFORMATION

The report was supported by Konica Minolta, Inc. (Tokyo, Japan).

## CONFLICT OF INTEREST

The authors have no conflicts of interest to declare.

## ETHICAL APPROVAL

Consent was obtained from patients.

## CONSENT

Written informed consent was obtained from the patient to publish this report in accordance with the journal's patient consent policy.

## Supporting information


Video S1

Video S2

Video S3

Video S4

## Data Availability

All data included in this report are accurate to the best of our knowledge. We will make available data (images and reports) upon request.
